# P-879. Antimicrobial Use Analysis in Tennessee from Healthcare-Associated Infections and Antimicrobial Use Prevalence Survey, 2023

**DOI:** 10.1093/ofid/ofaf695.1087

**Published:** 2026-01-11

**Authors:** Dipen Patel, Glodi Mutamba, Casey L Morrell, Christopher Wilson, Christopher D Evans, Melphine Harriott

**Affiliations:** HAI/AR, Tennessee Department of Health, Nashville, TN; Tennessee Department of Health, Nashville, Tennessee; Tennessee Department of Health, Nashville, Tennessee; Tennessee Department of Health, Nashville, Tennessee; Tennessee Department of Health, Nashville, Tennessee; TN Department of Health, Nashville, Tennessee

## Abstract

**Background:**

The Tennessee (TN) Department of Health's Healthcare-Associated Infections and Antimicrobial Resistance (HAI/AR) Program, in collaboration with nine other Emerging Infections Program sites, participated in the CDC’s 2023 Healthcare-Associated Infections and Antimicrobial Use Prevalence Survey (HPPS). This multistate initiative aimed to evaluate HAI prevalence and antimicrobial use (AU) patterns in U.S. acute care hospitals (ACH). This study uses TN HPPS data to investigate patient demographics and antimicrobial prescribing trends in participating TN hospitals and identifies opportunities for targeted antimicrobial stewardship (AS).

**Figure:**
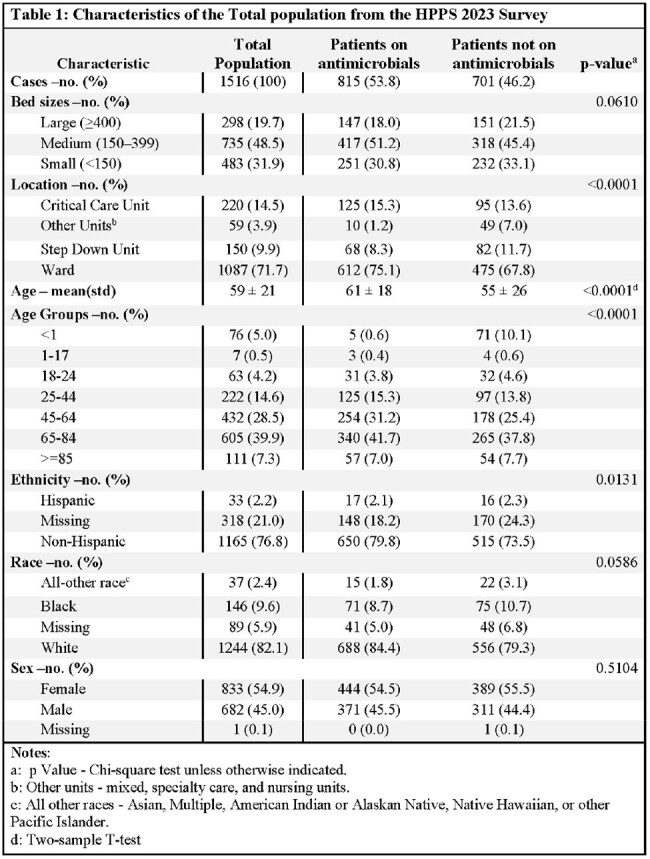

**Figure:**
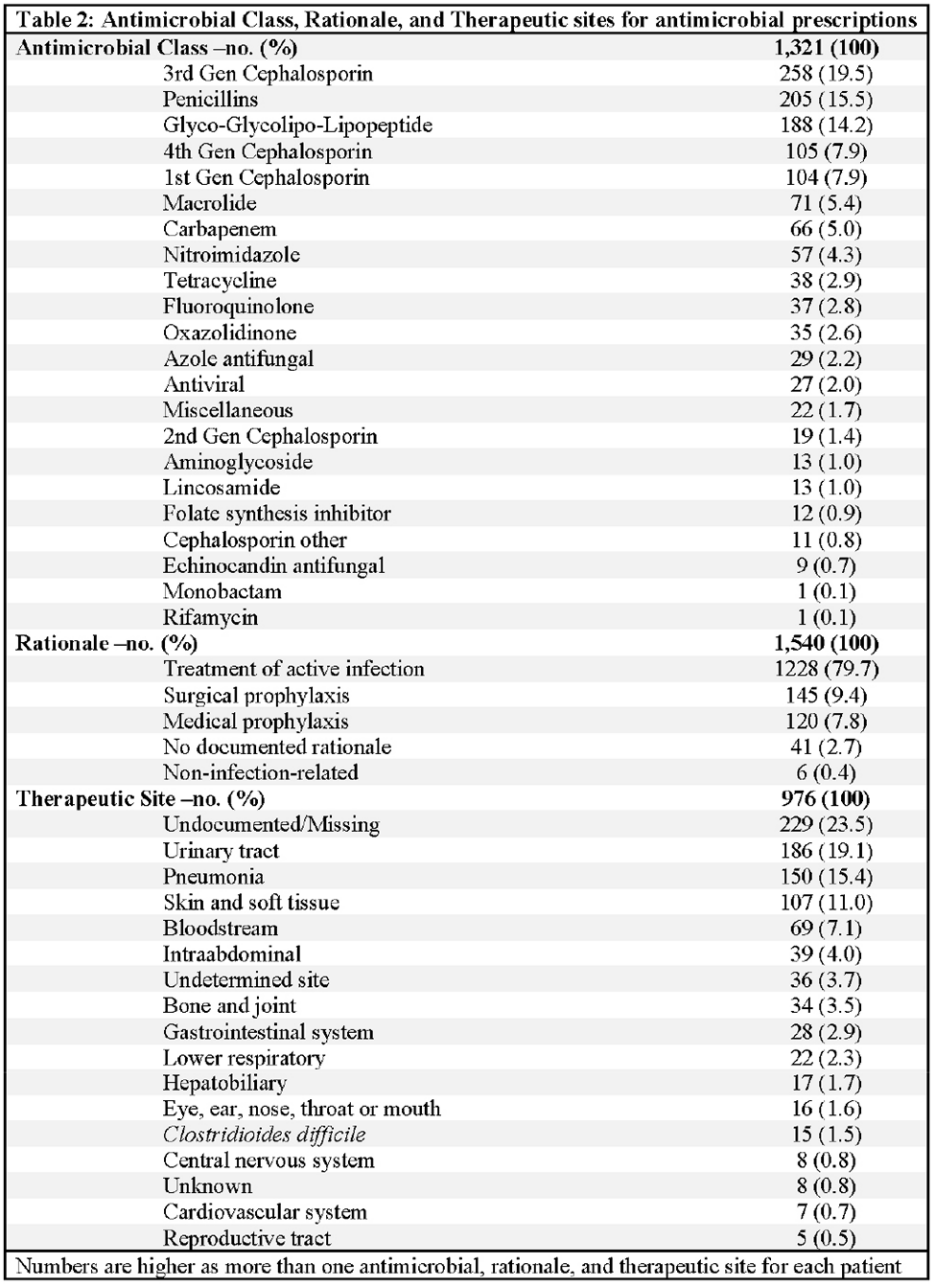

**Methods:**

Twenty-five ACHs in TN were selected for HPPS 2023 using CDC-defined site criteria and categorized by bed size: small (< 150), medium (150–399), or large (≥400). Randomized patient-level data were collected on survey dates. Demographics were compared between patients receiving antimicrobials and those not. Antimicrobial cases were analyzed by class, rationale (e.g., treatment of active infection, prophylaxis), and therapeutic site. Descriptive statistics, chi-square tests, and t-tests were performed using SAS v9.4.

**Results:**

Of the 1,516 patients included in the HPPS, 53.8% (815) received antimicrobials. The mean age of antimicrobial recipients was 61 (< 0.0001). Patients receiving antimicrobials were older adults (41.7% vs 37.8%; p=0.0001), non-Hispanic (79.8% vs 73.5%; p=0.0131), and were in the hospital ward unit (75.1% vs 67.8%; p < 0.0001) compared to non-recipients. Of 1,321 antimicrobial medications, the most common groups were third-generation cephalosporins (19.5%), penicillins (15.5%), and glycol-lipopeptides (14.2%). Most antimicrobials were prescribed for active infections (79.7%), and the leading therapeutic site was the urinary tract (19.1%).

**Conclusion:**

This analysis demonstrated a widespread AU pattern, with over half of the patients receiving at least one antimicrobial. Demographic variations were observed, with patients’ information playing a key role. Ongoing research should monitor facility-level trends and assess AS interventions. Broader implementation of surveys like HPPS can strengthen benchmarking and inform public health strategies to optimize AU and improve patient safety.

**Disclosures:**

All Authors: No reported disclosures

